# FAdV-4 without *Fiber-2* Is a Highly Attenuated and Protective Vaccine Candidate

**DOI:** 10.1128/spectrum.01436-21

**Published:** 2022-02-02

**Authors:** Quan Xie, Weikang Wang, Qiuqi Kan, Yaru Mu, Wei Zhang, Jian Chen, Luyuan Li, Hui Fu, Tuofan Li, Zhimin Wan, Wei Gao, Hongxia Shao, Aijian Qin, Jianqiang Ye

**Affiliations:** a Key Laboratory of Jiangsu Preventive Veterinary Medicine, Key Laboratory for Avian Preventive Medicine, Ministry of Education, College of Veterinary Medicine, Yangzhou University, Yangzhou, Jiangsu, China; b Jiangsu Co-innovation Center for Prevention and Control of Important Animal Infectious Diseases and Zoonoses, Yangzhou, Jiangsu, China; c Joint International Research Laboratory of Agriculture and Agri-Product Safety, The Ministry of Education of China, Yangzhou University, Yangzhou, Jiangsu, China; d Institutes of Agricultural Science and Technology Development, Yangzhou University, Yangzhou, Jiangsu, China; e Sinopharm Yangzhou VAC Biological Engineering Co. Ltd., Yangzhou, Jiangsu, China; University of Prince Edward Island

**Keywords:** FAdV-4, CRISPR/Cas9, deletion of *fiber-2*, virus rescuing, pathogenesis, protection

## Abstract

Hepatitis-hydropericardium syndrome (HHS) caused by the highly pathogenic fowl adenovirus serotype 4 (FAdV-4) has resulted in huge economic losses to the poultry industry globally. The *fiber-2* gene, as a major virulence determiner, is also an important vaccine target against FAdV-4. In this study, we used a CRISPR/Cas9-based homology-dependent recombinant technique to replace the *fiber-2* gene with *egfp* and generate a novel recombinant virus, designated FAdV4-EGFP-rF2. Although FAdV4-EGFP-rF2 showed low replication ability compared to the wild-type FAdV-4 in LMH cells, FAdV4-EGFP-rF2 could effectively replicate in LMH-F2 cells with the expression of Fiber-2. Moreover, FAdV4-EGFP-rF2 was not only highly attenuated in chickens, but also could provide efficient protection against a lethal challenge of FAdV-4. Moreover, FAdV4-EGFP-rF2 without *fiber-2* could induce neutralizing antibodies at the same level as FA4-EGFP with *fiber-2*. These results clearly demonstrate that although *fiber-2* affects the viral replication and pathogenesis of FAdV-4, it is not necessary for virus replication and induction of neutralizing antibodies; these findings provide novel insights into the roles of *fiber-2* and highlight *fiber-2* as an insertion site for generating live-attenuated FAdV-4 vaccines against FAdV-4 and other pathogens.

**IMPORTANCE** Among all serotypes of fowl adenovirus, serotypes FAdV-1, FAdV-4, and FAdV-10 are unique members with two *fiber* genes (*fiber-1* and *fiber-2*). Recent studies reveal that Fiber-1, not Fiber-2, directly triggers viral infection of FAdV-4, whereas Fiber-2, but not Fiber-1, has been identified as the major virulence determiner and an efficient protective immunogen for subunit vaccines. Here, we replaced *fiber-2* with *egfp* to generate a novel recombinant virus, designated FAdV4-EGFP-rF2. *In vitro* and *in vivo* studies on FAdV4-EGFP-rF2 revealed that *fiber-2* was not necessary for either virus replication or efficient protection for FAdV-4; these results not only provide a novel live-attenuated vaccine candidate against HHS, but also give new ideas for generating a FAdV-4 based vaccine vector against other pathogens.

## INTRODUCTION

Fowl adenoviruses (FAdVs) are nonenveloped viruses with double-stranded DNA, belonging to the family *Adenoviridae* and genus *Aviadenovirus (*1). Based on restriction enzyme digestion patterns and serum cross-neutralization tests, FAdVs are divided into 5 species (FAdV-A to FAdV-E) with 12 serotypes (FAdV-1 to 8a and 8b to 11) (2). The recent outbreaks of hepatitis-hydropericardium syndrome (HHS) in chickens around the world are mainly caused by the highly pathogenic serotype 4 fowl adenovirus (FAdV-4). Unlike other serotypes of fowl adenovirus, serotypes FAdV-1, FAdV-4, and FAdV-10 have two Fiber proteins on the viral surface, designated Fiber-1 and Fiber-2 (3). Fiber-1 and Fiber-2 play vital roles in mediating the infection and pathogenesis of FAdV-4, respectively. Recently, studies by our group and other groups found that Fiber-1, not Fiber-2, directly triggered the viral infection of FAdV-4 (4, 5), whereas Fiber-2 and Hexon, but not Fiber-1, were the virulence determiners for the highly pathogenic FAdV-4 in comparison with the nonpathogenic strain (6–8). Notably, as one of the capsid proteins of FAdV-4, Fiber 2 has been identified as an efficient protective immunogen for subunit vaccine candidates (9). Many studies have proven that the recombinant Fiber-2 protein can provide better protection against lethal challenge from FAdV-4 than other capsid proteins, including Fiber-1, Penton, and Hexon (9–13). However, Fiber-2 protein cannot induce detectable neutralizing antibodies against FAdV-4 (9). It is also worth noted that the inactivated FAdV-4 vaccine candidates can induce robust neutralizing antibodies and provide efficient protection (14). These results indicate that although Fiber-2 plays a vital role in the pathogenesis of FAdV-4 and can provide efficient protection, Fiber-2 might not contribute to the induction of neutralizing antibody by inactivated FAdV-4. In addition, the generation of the recombinant virus FA4-EGFP, with EGFP fused with Fiber-2, indicates that Fiber-2 can be edited (8). Therefore, Fiber-2 may be as a potential target for knockout to generate live-attenuated FAdV-4 vaccine candidates or recombinant vaccine vectors.

To test our hypothesis, we constructed a donor plasmid with *egfp* replacing *fiber-2* and generated a recombinant virus without *fiber-2*, designated FAdV4-EGFP-rF2, using the CRISPR-Cas9 technique. *In vitro* and *in vivo* studies revealed that FAdV4-EGFP-rF2 was not only highly attenuated, but could also provide efficient protection against FAdV-4 with robust neutralizing antibody.

## RESULTS

### Generation and identification of the recombinant virus FAdV4-EGFP-rF2 without *fiber-2*.

**FIG 1:**
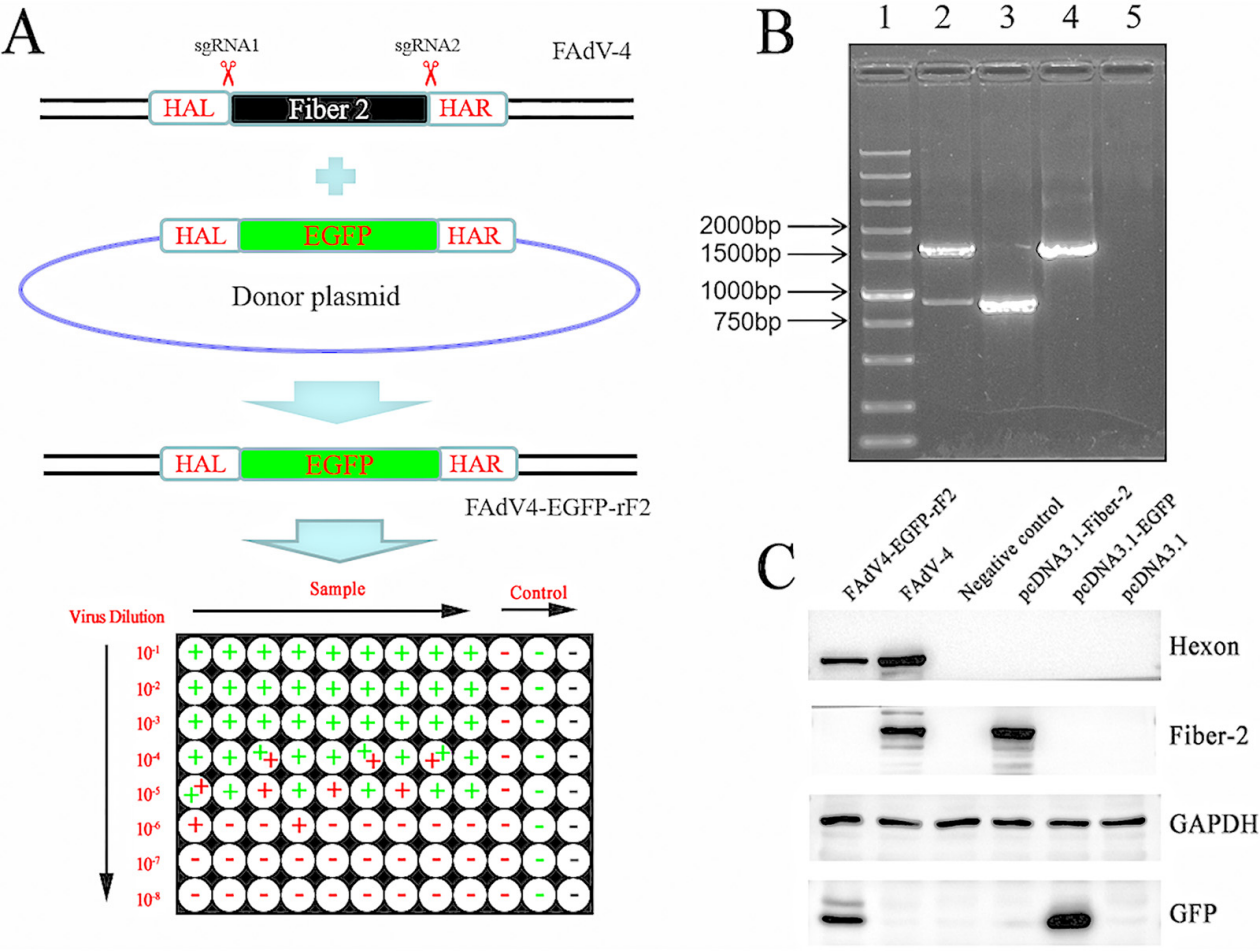
Generation and identification of the recombinant virus FAdV4-EGFP-rF2. (A) Strategy of the homology-dependent recombinant for generating FAdV4-EGFP-rF2 using a CRISPR/Cas 9 system. LMH cells were first transfected with sgRNA1 and sgRNA2. At 24 hpt (hours posttransfection), the LMH cells were infected with FAdV-4 and transfected with donor plasmid. The rescued FAdV4-EGFP-rF2 was then purified by limiting dilution assay. (B) PCR identification of FAdV4-EGFP-rF2. The DNA of unpurified FAdV4-EGFP-rF2 (lane 2), purified FAdV4-EGFP-rF2 (lane 3), and wild-type (WT) FAdV-4 (lane 4) was extracted and detected by PCR using specific primers. Lane 1 shows the DNA marker and lane 5 shows the negative control. (C) LMH cells infected with purified FAdV4-EGFP-rF2 and wild-type FAdV-4, or transfected with pcDNA3.1-Fiber-2 and pcDNA3.1-EGFP, respectively, were harvested at the indicated time points and lysed; the lysates were then detected by monoclonal antibodies against Fiber-2, Hexon, GFP, and GAPDH.

To test whether the *fiber-2* gene could be knocked out in FAdV-4, sgRNAs targeting the N- and C terminus of *fiber-2* were designed and subsequently cloned into lentiCRISPR v2. In our strategy (Fig. 1A), the donor plasmid with *egfp* replacing *fiber-2* was constructed and transfected into leghorn male hepatoma (LMH) cells with sgRNA transfection and viral infection of FAdV-4. The recombinant virus FAdV4-EGFP-rF2 was then purified through serial limit dilution and immunofluorescence assay (IFA), and the purified FAdV4-EGFP-rF2 was further identified by sequencing and PCR using the primers listed in Table 1. As shown in Fig. 1B, two bands indicating FAdV-4 and FAdV4-EGFP-rF2 could be amplified in the unpurified sample, while only a single specific band could be amplified in either FAdV-4 or the purified FAdV4-EGFP-rF2 virus. In the Western blot analysis (Fig. 1C), Fiber-2 and Hexon could be efficiently detected in the LMH cells infected with FAdV-4, whereas the Fiber-2 could be not detected in LMH cells infected with FAdV4-EGFP-rF2. Instead, EGFP and Hexon could be efficiently detected in the LMH cells infected with FAdV4-EGFP-rF2. All these data demonstrated that a novel recombinant virus FAdV4-EGFP-rF2 without *fiber-2* was generated, highlighting that *fiber-2* is not necessary for the viral replication of FAdV-4.

**TABLE 1:** PCR primers for constructing donor plasmid and detecting recombinant virus

PCR product	Primer sequence (5′–3′)
HAL	F: GGTGACCTACTGACCCTCAACACC
	R: GAACAGCTCCTCGCCCTTGCTCACCATTGTTCCCGTTGGGGGA
HAR	F: CATGGACGAGCTGTACAAGTAAGCGCGCCCTCCCCACCGCGTG
	R: CTACTTTACCTGCATTTCGTCAG
EGFP	F: CTACTCCCCCAACGGGAACAATGGTGAGCAAGGGCGAGG
	R: GGAGGGCGCGCTTACTTGTACAGCTCGTCCATGCCGAGAG
Partial *fiber-2*	F: CTCCAACTGGTTTGACCAGAACG
	R: GTTGTATGATTGGACGCGGGAAC

### FAdV4-EGFP-rF2 replicated inefficiently in LMH, but efficiently in LMH-F2.

**FIG 2:**
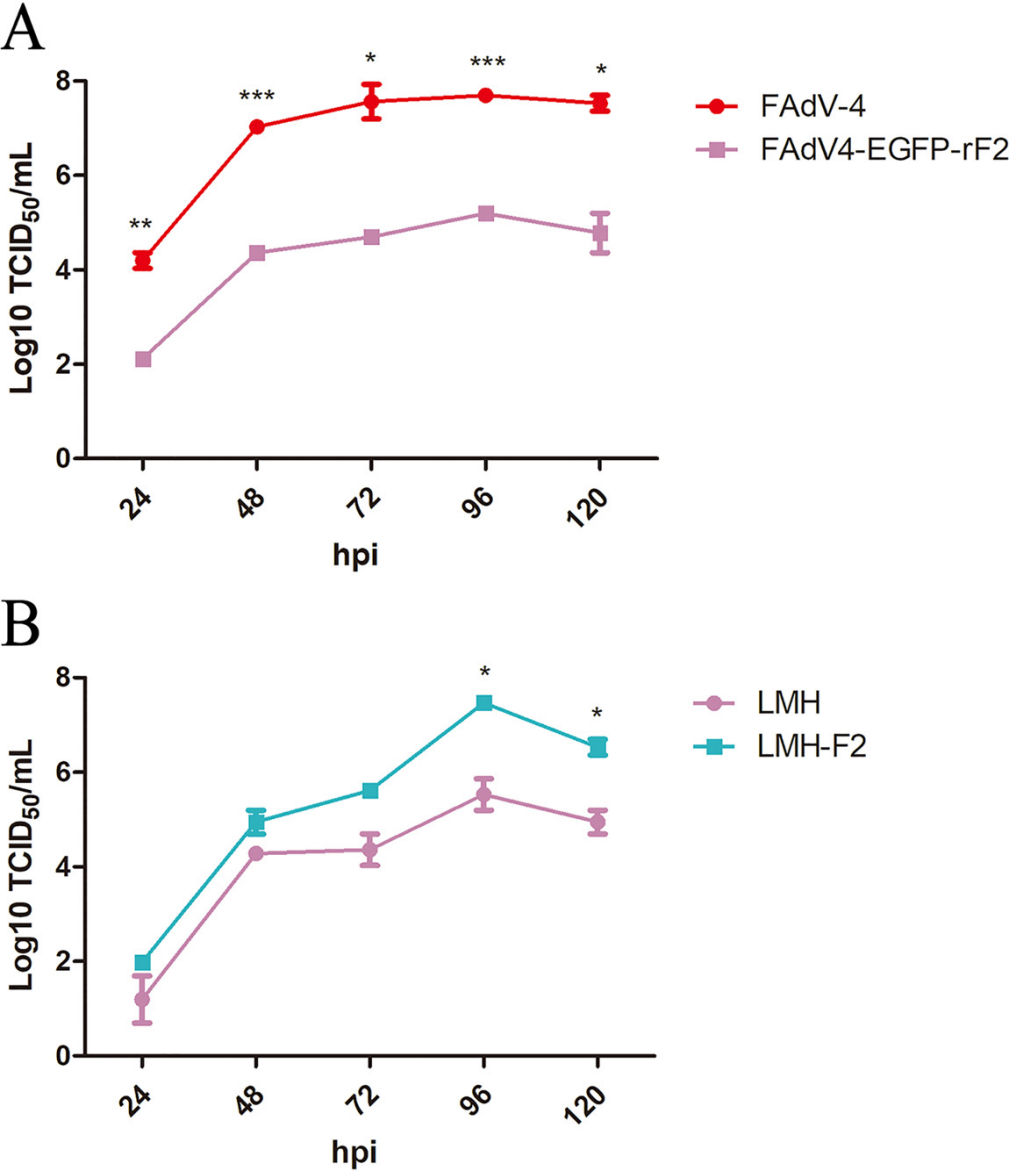
FAdV4-EGFP-rF2 replicated inefficiently in LMH, but efficiently in LMH-F2. (A) LMH cells were infected with FAdV4-EGFP-rF2 and FAdV-4 at an MOI of 0.1, respectively; viral supernatants collected from infected LMH cells at the indicated time points were then titrated by TCID_50_. (B) LMH and LMH-F2 cells were infected with FAdV4-EGFP-rF2 at an MOI of 0.1, respectively, and the viral supernatants collected from infected cells at the indicated time points were then titrated by TCID_50_. All tests were done in triplicate and repeated twice. Statistical analysis in this experiment was performed with a Student’s *t* test using GraphPad 5 software.

To determine the growth kinetics of the novel virus FAdV4-EGFP-rF2, LMH cells were infected with FAdV-4 and FAdV4-EGFP-rF2, respectively, and the viral supernatants were harvested for viral titration at the indicated time points. As shown in Fig. 2A, FAdV4-EGFP-rF2 replicated much slower than FAdV-4 in LMH cells. The peak titer of FAdV4-EGFP-rF2 in LMH cells was about 500-fold lower than that of FAdV-4, and the highest titer of FAdV4-EGFP-rF2 was only 10^5^ TCID_50_ (50% tissue culture infective dose)/mL. However, FAdV4-EGFP-rF2 could replicate efficiently in the LMH cells which stably expressed the Fiber-2 protein (designated LMH-F2 cells). As described in Fig. 2B, the peak titer of FAdV4-EGFP-rF2 could reach 10^7^ TCID_50_/mL in the LMH-F2 cell lines, which was 100-fold higher than that in LMH cells. These results demonstrate that although the replication capacity of FAdV4-EGFP-rF2 is much weaker than that of FAdV-4, FAdV4-EGFP-rF2 can efficiently replicate in LMH-F2 cells which stably express the Fiber-2 protein, supporting the replication of FAdV4-EGFP-rF2 with a higher titer.

### FAdV4-EGFP-rF2 without *fiber-2* was highly attenuated *in vivo*.

**FIG 3:**
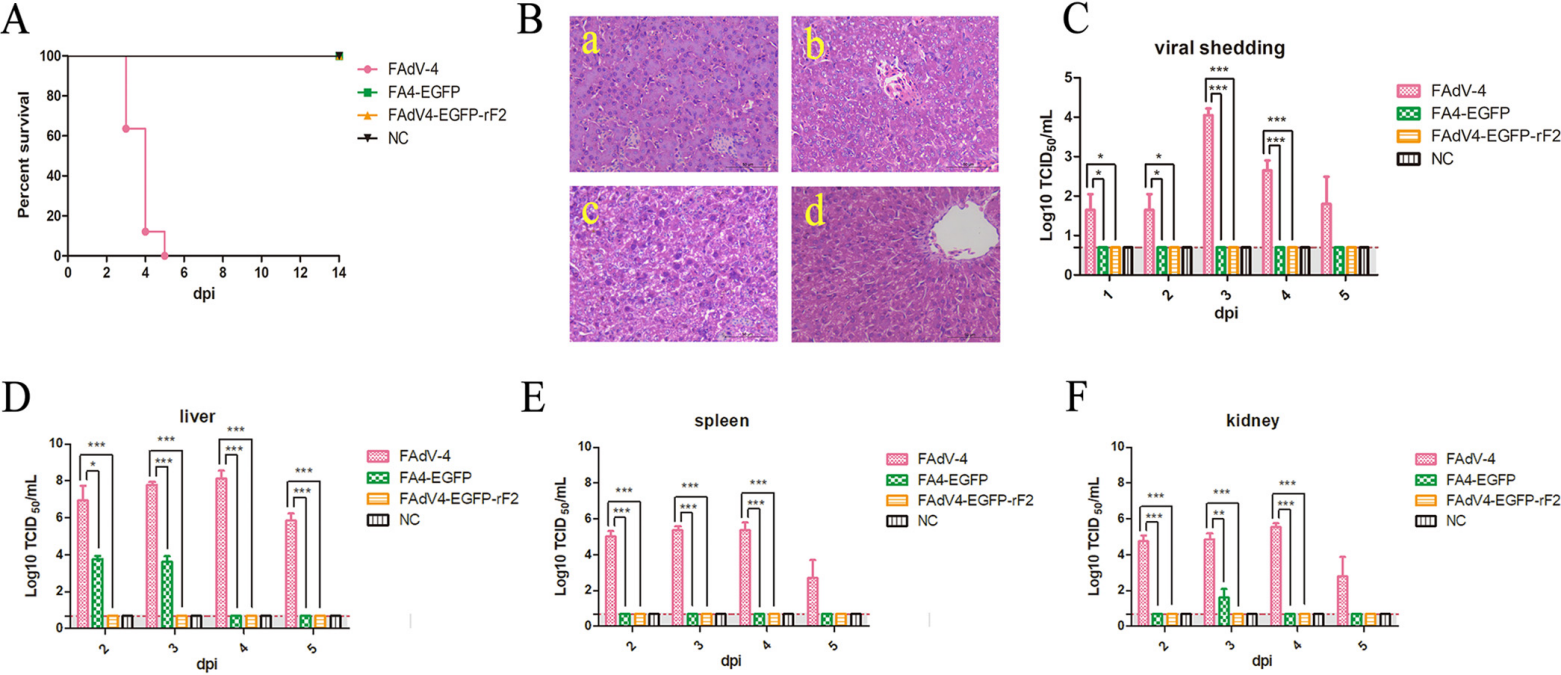
FAdV4-EGFP-rF2 without *fiber-2* was highly attenuated *in vivo.* A total of 160 2-week-old SPF chickens were randomly divided into 4 groups (*n* = 40). Chickens in group I were inoculated with WT FAdV-4, while those in groups II and III were inoculated with FA4-EGFP and FAdV4-EGFP-rF2, respectively. All chickens were intramuscularly inoculated with 2.5 × 10^4^ TCID_50_ of indicated virus in 200 μL of culture medium containing 1% FBS, and those in group IV, inoculated with the same volume of culture medium containing 1% FBS, were set as a negative control. Morbidity and mortality were recorded daily. Cloacal swabs and organ tissues from each group were collected at indicated time points for viral titration. (A) Percent survival for infected chickens. (B) Representative histological changes in liver tissues from chickens inoculated with FAdV4-EGFP-rF2 (yellow), FA4-EGFP (green), wild-type FAdV-4 (red), and negative control (black). (C) Viral shedding in the infected chickens. Viral loads in liver (D), spleen (E), and kidney (F) from the infected chickens. Statistical analysis in this experiment was performed with one-way ANOVA test using GraphPad 5 software.

To evaluate the pathogenicity of FAdV4-EGFP-rF2, chickens randomly divided into 4 groups were intramuscularly inoculated with indicated virus or culture medium containing 1% fetal bovine serum (FBS). The clinical symptoms and mortality of the infected chickens were monitored daily. As a result, the chickens inoculated with FAdV-4 all died within 5 days postinfection (dpi), and typical symptoms could be observed after necropsy; however, chickens inoculated with FAdV4-EGFP-rF2 and FA4-EGFP did not die or develop any clinical signs (Fig. 3A). In addition, histopathological analysis showed that the FAdV-4 group chickens presented severe inclusion body hepatitis (IBH), degeneration, and necrosis in liver tissues, whereas the FAdV4-EGFP-rF2 and FA4-EGFP groups were similar to the negative-control group (Fig. 3B). For viral shedding, as shown in Fig. 3C, high viral titers of about 10^3^ to 10^4^ TCID_50_/mL were detected in cloacal swabs from chickens infected with FAdV-4 at 2 to 3 dpi, whereas no virus was detected in cloacal swabs from chickens in the FA4-EGFP or FAdV4-EGFP-rF2 groups. For the viral loads in tissues, the liver, spleen, and kidney were collected at 2, 3, 4, and 5 dpi, respectively. As shown in Fig. 3D, the viral loads in livers from the FAdV-4 group reached 10^7^ to 10^8^ TCID_50_/mL within 2 to 4 dpi, while those in the FA4-EGFP group were significantly lower, only 10^3^ to 10^4^ TCID_50_/mL at 2 to 3 dpi. Surprisingly, viral loads in livers from the FAdV4-EGFP-rF2 group were not detectable. Similar results were found in the spleen and kidney (Fig. 3E to F). High-level titers were detected in the FAdV-4 group, while those in the FA4-EGFP group were barely detected, and those in the FAdV4-EGFP-rF2 group were not detectable. Collectively, our results demonstrate that FAdV4-EGFP-rF2 without *fiber-2* was highly attenuated *in vivo*.

### Fiber-2 did not contribute to the induction of neutralizing antibody.

**FIG 4:**
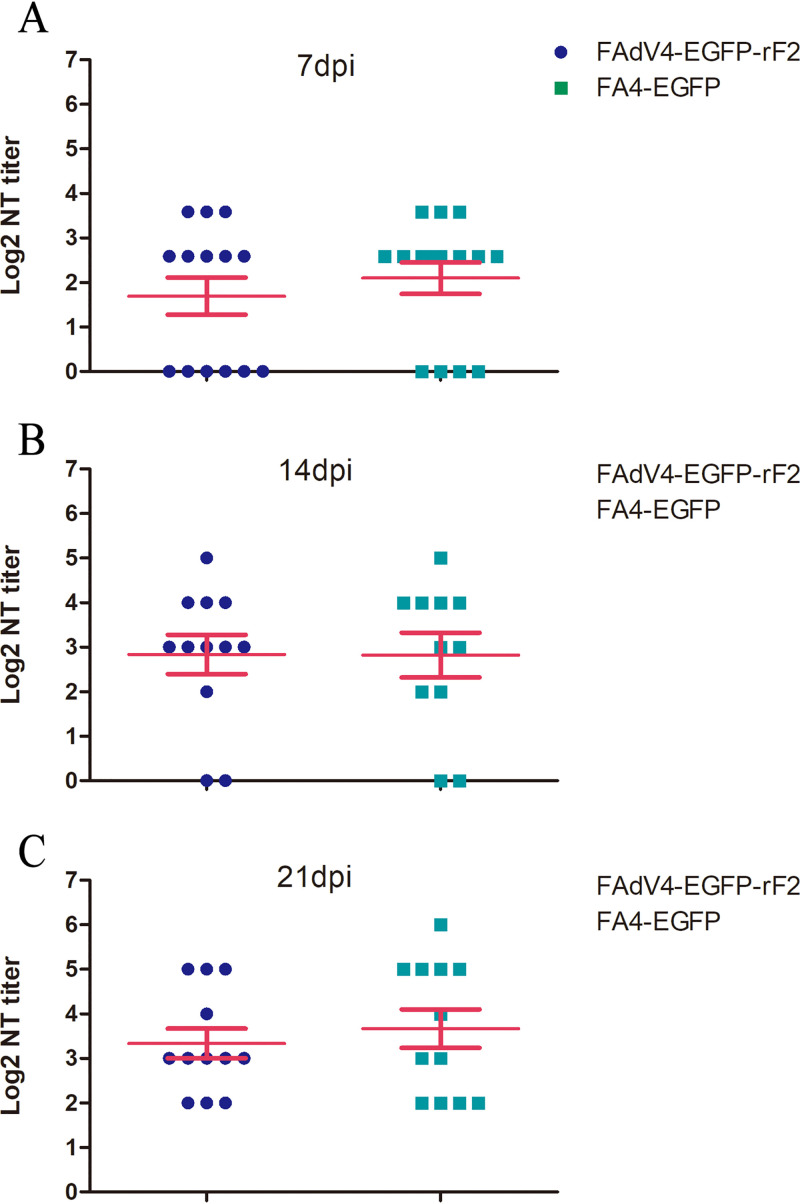
Fiber-2 did not contribute to the induction of neutralizing antibody. Chicken sera were collected from chickens inoculated with FA4-EGFP (group II) and FAdV4-EGFP-rF2 (group III), respectively, at the indicated time points, and tested for titers of neutralizing antibodies. Panels A, B, and C show NT titers at 7, 14, and 21 dpi, respectively.

To determine the capacity of Fiber-2 to induce neutralizing antibodies, chickens in group II were inoculated with FA4-EGFP carrying the full-length *fiber-2*, and chickens in group III were inoculated with FAdV4-EGFP-rF2 without *fiber-2*. The sera were collected at the indicated time points and tested for neutralizing antibody. As described in Fig. 4, the average neutralization test (NT) titers of sera in chickens from the FA4-EGFP group were 1.96, 2.81, and 3.67 at 7, 14, and 21 dpi, respectively, whereas those from the FAdV4-EGFP-rF2 group were 1.69, 2.83, and 3.33 correspondingly. No significant difference was found between the two groups, indicating that Fiber-2 is not necessary for inducing neutralizing antibodies against FAdV-4.

### FAdV4-EGFP-rF2 provided efficient protection against FAdV-4 challenge.

**FIG 5:**
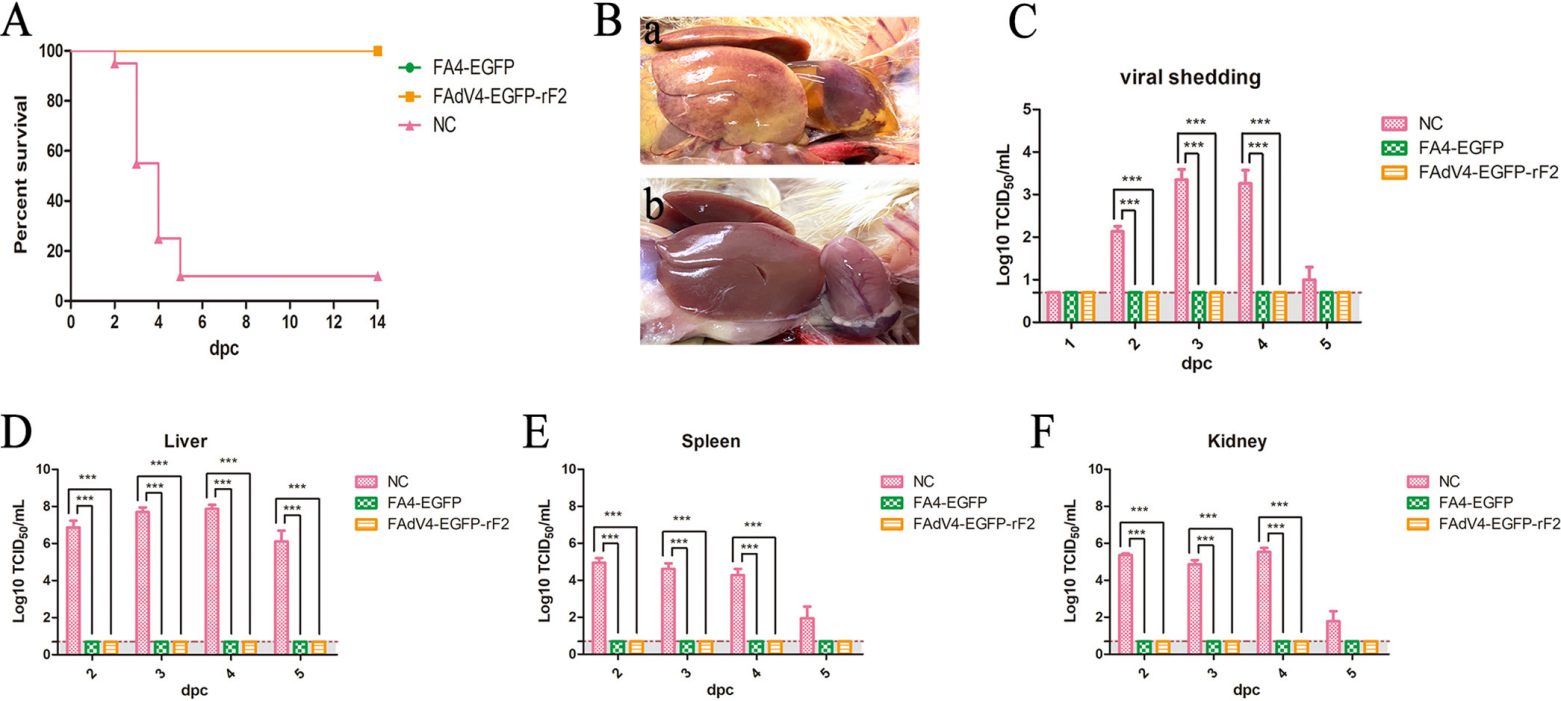
FAdV4-EGFP-rF2 provided efficient protection against FAdV-4 challenge. The survival chickens inoculated with FAdV4-EGFP-rF2, FA4-EGFP, and culture medium containing 1% FBS were challenged with a lethal dose of FAdV-4 at 21 dpi. The morbidity and mortality were recorded daily. Cloacal swabs and organ tissues from each group were collected at the indicated time points for viral titration. (A) Percent survival of the challenged chickens. (B) Representative gross lesions in heart and liver from negative-control chickens (top) and chickens previously inoculated with FAdV4-EGFP-rF2 (bottom) after challenge. (C) Viral shedding in cloacal swabs from challenged chickens. Viral loads in liver (D), spleen (E), and kidney (F) tissues from challenged chickens. Statistical analysis in this experiment was performed with a one-way ANOVA test using GraphPad 5 software.

To further evaluate the protective efficacy of FAdV4-EGFP-rF2, chickens in group II, group III and the negative-control group were challenged with a lethal dose of FAdV-4 by intramuscular injection. The clinical symptoms and mortality of the challenged chickens were monitored daily. As shown in Fig. 5A, the chickens in the control group began to die at 2 days postchallenge (dpc) and finally reached 90% mortality within 5 dpc. After challenge, the chickens in the control group showed signs of depression, loss of appetite, yellow-green and thin feces, and huddling together with ruffled feathers. Moreover, the typical lesions of HHS could be easily observed after necropsy for the chickens in the control group. In contrast, the chickens previously inoculated with FA4-EGFP and FAdV4-EGFP-rF2 did not show any signs of HHS and all survived after the challenge (Fig. 5B). To detect viral titers, cloacal swabs from each group were collected at 1, 2, 3, 4, and 5 dpc, and the liver, spleen, and kidney were collected at 2, 3, 4, and 5 dpc. As described in Fig. 5C to F, no virus was detected in the cloacal swabs or organ tissues from chickens in groups II and III, which had been previously inoculated with FA4-EGFP or FAdV4-EGFP-rF2, while high viral titers were detected in the organ tissues and cloacal swabs in from the control group. These data demonstrate that FAdV4-EGFP-rF2 without *fiber-2* can provide efficient protection against FAdV-4 challenge.

## DISCUSSION

The recent outbreak of HHS caused by the highly pathogenic FAdV-4 has significantly restricted the sustainable development of the global poultry industry (15–17). Although several vaccines have been developed to control the diseases of FAdV-4, the molecular pathogenesis and protective basis for FAdV-4 need to be further elucidated. As two important antigens in the viral capsid, both Fiber-1 and Fiber-2 are thought to play vital roles in the viral infection and pathogenesis of FAdV-4. Fiber-1 has been identified as a viral ligand which interacts with the cell receptor CAR-homology to directly initiate FAdV-4 infection (4, 5), whereas Fiber-2 has been identified as one of the virulence determiners (6, 8). Notably, although the recombinant Fiber-2 protein cannot induce detectable neutralizing antibody, Fiber-2 protein can provide better protection against FAdV-4 than other capsid proteins, including Fiber-1, Penton, and Hexon (9–13).

In this study, we used the CRISPR/Cas 9 technique to replace the *fiber-2* with *egfp* and generated a recombinant virus, FAdV4-EGFP-rF2, which lacks *fiber-2. In vivo* data reveal that FAdV4-EGFP-rF2 is not only highly attenuated, but can also provide efficient protection against FAdV-4 with neutralizing antibody, highlighting that Fiber-2 is unnecessary not only for viral replication, but also for efficient protection against FAdV-4. Although viral load was not detected in the organ tissues by TCID_50_, the virus could be detected by PCR (data not shown), indicating that FAdV4-EGFP-rF2 replicated at a very low level *in vivo*. These results suggest that, except for Fiber-2, other proteins (such as Fiber-1 and Hexon) should play vital roles in inducing efficient protection against FAdV-4 infection. The finding that FAdV4-EGFP-rF2 without *fiber-2* can induce neutralizing antibodies also reveals that *fiber-2* does not play a vital role in producing neutralizing antibodies, highlighting the need to elucidate the molecular basis for the protection provided by subunit Fiber-2 vaccines. More recently, Luca et al. showed that the Fiber protein of FAdV-8a could induce neutralizing antibody and provide protection against a homotypic challenge (18). Although previous studies have provided evidence that the Fiber-1 protein or its knob domain could induce neutralizing antibody against FAdV-4 (4, 5), the capacity of the Fiber-1 of FAdV-4 to induce efficient neutralizing antibody against FAdV-4 in chickens also needs to be further investigated.

It should be mentioned that we could not rescue the recombinant virus with replacement of *fiber-1* with *egfp* by the same CRISPR/Cas9 technique, indicating that *fiber-1* of FAdV-4 is critical for virus assembly and infection; this is consistent with previous reports that *fiber-1* directly triggers FAdV-4 infection via cell receptor CAR-homology (4, 5). On the contrary, in the case of serotype FAdV-1, although Fiber-1 of CELO is responsible for its high affinity with the cell receptor CAR, CELO with a disrupted Fiber-1, but not with a disrupted Fiber-2, can be efficiently generated (19), suggesting different roles of Fibers between FAdV-1 and FAdV-4. In addition, the roles of Fiber-1 and Fiber-2 of FAdV-1 in protection against FAdV-1 need to be further elucidated.

The generation of recombinant viruses with deletion of virulence factors is a good strategy for developing live-attenuated vaccines, such as those against Marek’s disease virus (20) and pseudorabies virus (21). Recently, many studies revealed that DNA viruses with large genomes could be efficient viral vectors for delivering foreign genes (22, 23). FAdV-4, with a genome of about 45 kb, could provide multiple sites for foreign gene insertion. Our previous studies revealed that the highly pathogenic FAdV-4, recently isolated in China, carried a natural large genomic deletion (1,966 bp) between ORF 42 and 43 compared with the nonpathogenic strain ON1 (15). Pan et al. reported that foreign genes inserted into this deletion site did not affect viral replication or pathogenesis (24). Therefore, this deletion site may be suitable for developing inactivated recombinant vaccines, but it is unsuitable for developing live-attenuated recombinant vaccines. In contrast, since the recombinant virus FAdV4-EGFP-rF2 without *fiber-2*, generated here, is highly attenuated and can provide efficient protection with neutralizing antibodies, *fiber-2* can serve as an efficient foreign gene insertion site for generating FAdV-4-based, live-attenuated vaccines to protect against FAdV-4 and other pathogens.

It should be also mentioned that Schonewille et al. generated the first live-attenuated FAdV-4 through continuous passages in QT35 cells (25). Although FAdV-4/QT35 cannot induce detectable neutralization antibodies through oral inoculation, FAdV-4/QT35 can provide full protection against a lethal challenge of FAdV-4 (26). Since oral vaccination is obviously a prior option for mass application, the protective efficacy of oral inoculation of FAdV4-EGFP-rF2 needs to be further tested. Unlike the host adaptation strategies for developing live-attenuated FAdV-4 vaccine candidates used by Schonewille et al., the CRISPR/Cas 9 and homological recombinant techniques used in this study are more controllable and specific for efficiently generating live-attenuated vaccines.

Notably, although the recombinant virus FAdV4-EGFP-rF2 replicated much slower than the wild-type FAdV-4 in LMH cells, it could replicate efficiently in LMH-F2 cells with high viral titers. Therefore, the cell line LMH-F2 can be used to efficiently amplify such recombinant viruses for vaccine development. Of course, whether the *egfp* gene in the recombinant virus FAdV4-EGFP-rF2 could be replaced by other foreign genes to generate FAdV-4-based, live-attenuated recombinant vaccines needs to be further evaluated.

## MATERIALS AND METHODS

### Cells, viruses, and antibodies.

The FAdV-4 strain SD2015 was isolated and stored in our laboratory (15), and propagated in leghorn male hepatoma (LMH) cells. The recombinant virus FA4-EGFP with *egfp* fused to the N terminus of full-length *fiber-2* was generated and stored in our laboratory (27). The recombinant virus FAdV4-EGFP-rF2 without *fiber-2*, which was replaced with *egfp*, was generated in this study and stored in our laboratory. The LMH cells were purchased from ATCC. The LMH-F2 cells stably expressing the Fiber-2 protein were generated and stored in our laboratory. Both LMH and LMH-F2 cells were cultured in Dulbecco’s Modified Eagle’s Medium (DMEM)/F12 (Gibco, NY, USA) supplemented with 10% FBS (Lonsera, Shanghai, China) in 5% CO_2_ incubator at 37°C. Monoclonal antibody (MAb) 3B5 against Fiber-1 and MAb 3C2 against Fiber-2 were generated in our laboratory; MAb 1C9 against Fiber-2 and MAb 1B5 against Hexon were kindly provided by Hongjun Chen.

### Construction of sgRNAs and donor plasmids.

The sgRNAs targeting the FAdV-4 Fiber 2 were designed using CRISPR guide RNA designing website (www.benchling.com) and cloned into the CRISPR-Cas9 vector lentiCRISPR v2. The sequences of the sgRNAs are listed in Table 2. The donor plasmid with *egfp* replacing *fiber-2* was constructed by overlap-PCR. The *egfp* gene and the 1,000-bp homologous arm (HA) flanking the *fiber-2* gene were amplified and assembled as HAL-EGFP-HAR, and finally cloned into the pMD19 simple vector. The primers used for constructing the donor plasmid are listed in Table 1.

**TABLE 2:** List of primers used for sgRNA cloning

sgRNA	Primer sequence (5′–3′)
sgRNA1	F: CACCGGGTTTATCCTTTCGATTACG
	R: AAACCGTAATCGAAAGGATAAACCC
sgRNA2	F: CACCGCGTGCTCTACAGCTGTCCAG
	R: AAACCTGGACAGCTGTAGAGCACGC

### Generation of the recombinant virus FAdV4-EGFP-rF2 without *fiber-2*.

LMH cells were first transfected with 2 μg of sgRNAs targeting the *fiber-2* gene. At 24 h posttransfection, LMH cells were infected with FAdV-4 at an MOI of 0.1, and immediately transfected with 4 μg of the donor plasmid. The recombinant virus, designated FAdV4-EGFP-rF2, was then purified by limiting dilution assay and indirect immunofluorescence assay (IFA), and the purified virus was further identified by Western blot, PCR, and sequencing.

### Growth curve of the FAdV4-EGFP-rF2.

To determine the growth kinetics of FAdV4-EGFP-rF2, LMH and LMH-F2 cells seeded in a 6-well plate (about 1.2 × 10^6^ cells per well) were infected with wild type FAdV-4 and the recombinant virus FAdV4-EGFP-rF2 at an MOI of 0.1, respectively. The viral supernatants were harvested at 24, 48, 72, 96, and 120 hpi (hours postinfection), and stored at −80°C until used. The TCID_50_ of the harvested viruses were determined in 96-well plates by serial dilution from 10^−1^ to 10^−8^ in triplicate. The diluted viruses were then inoculated into LMH cells immediately. At 96 hpi, LMH cells were fixed and detected by IFA, and viral titers were then calculated by the Reed-Muench method.

### Western blot assay.

LMH cells were seeded in a 12-well plate, and either infected with FAdV-4 and FAdV4-EGFP-rF2 at an MOI of 0.1, or transfected with 2 μg of pcDAN3.1-Fiber-2 and pcDNA3.1-EGFP, respectively. Then, LMH cells were collected at 2 dpi or 2 day posttransfection (dpt), and lysed in lysis buffer (CST, MA, USA) with proteolytic protease and phosphatase inhibitor cocktail (NCM, Soochow, China). The lysates were boiled in the loading buffer and then subjected to 10% SDS-PAGE and transferred to nitrocellulose (NC) membranes (GE Healthcare Life Sciences, Freiburg, Germany). After blocking with NCMblot blocking buffer (NCM, Soochow, China) for 0.5 h at room temperature (RT), the membranes were reacted with the corresponding antibodies for 1 h at RT. After being washed three times with phosphate-buffered saline with Tween 20, the membrane was incubated with horseradish peroxidase-labeled secondary antibodies for another 1 h at RT. After three washes, the membranes were developed with chemiluminescent reagents and imaged with an automatic imaging system (Tanon 5200).

### Immunofluorescence assay.

The LMH cells infected with viruses were fixed with a pre-chilled acetone:ethanol (3:2 vol/vol) mixture for 5 min at RT and then washed with PBS. The cells were then incubated with diluted MAb 3B5 against Fiber-1 for 45 min at 37°C. After being washed three times with PBS, the cells were incubated with the diluted second antibody (goat anti-mouse IgG-FITC) for another 45 min at 37°C. After three washes with PBS, the cells were observed by inverted fluorescence microscopy.

### Animal experiments.

A total of 160 2-week-old specific pathogen-free (SPF) chickens were randomly divided into 4 groups (*n* = 40). Chickens in group I were inoculated with wild-type (WT) FAdV-4, while those in groups II and III were inoculated with FA4-EGFP and FAdV4-EGFP-rF2, respectively. All chickens were intramuscularly inoculated with 2.5 × 10^4^ TCID_50_ of the indicated virus in 200 μL of culture medium containing 1% FBS, and those in group IV, inoculated with the same volume of culture medium containing 1% FBS, were set as a negative control. After infection, cloacal swabs were collected from five chickens in each group at the indicated time points to detect viral shedding; to titrate viral loads in different tissues, three chickens in each group were euthanized and necropsied at thee indicated time points. At 7, 14, and 21 days postinfection (dpi), the sera were collected for detection of neutralizing antibodies. At 21 dpi, all the survival chickens were challenged with 10^6^ TCID_50_ of FAdV-4 in 200 μL of culture medium containing 1% FBS by intramuscular inoculation. After challenge, three chickens in each group were euthanized and necropsied at the indicated time points, and cloacal swabs and organ tissues from each group were collected and tested for viral titration. The clinical symptoms and mortality of the challenged chickens were monitored daily. All animal experiments were performed in accordance with the Guidelines for Experimental Animals and protocol (SYXY-19) approved by the Animal Care and Use Committee of Yangzhou University (Yangzhou, China). At the end of the experiment, all the chickens were euthanized by CO_2_.

### Neutralization test.

First, the chicken sera were serially diluted, mixed with 100 TCID_50_ of FAdV-4 in 100 μL of culture medium containing 1% FBS, and incubated for 1 h at 37°C. After the LMH cells were washed once with PBS, the mixtures were added to a 96-well plate and incubated for 2 h at 37°C. After being washed once with PBS, the LMH cells were maintained in F12/DMEM medium containing 1% FBS. After being cultured for 96 h, the cells were fixed and subjected to IFA analysis using MAb 3B5 against Fiber-1 of FAdV-4, as previously (27).

### Statistical analysis.

All the results are presented as means ± standard deviation. The statistical analysis in this study was performed with a Student’s *t* test or a one-way ANOVA test using GraphPad 5 software. A *P* value of <0.05 was considered significant. *, **, and *** indicate *P* values less than 0.05, 0.01, and 0.001, respectively.

### Data availability.

The data sets used and/or analyzed during the current study are available from the corresponding author on reasonable request.
